# Caffeine consumption patterns, motivations, and adverse effects among Brazilian esports players: a cross-sectional study

**DOI:** 10.1080/15502783.2025.2579815

**Published:** 2025-11-26

**Authors:** Ellis Wollis Malta Abhulime, Heloisa Castanheira Santo André, Júlia Formagio, Bryan Saunders, Fabiana Braga Benatti

**Affiliations:** a University of Campinas (UNICAMP), School of Applied Sciences (FCA), Multidisciplinary Laboratory in Food and Health (LabMAS), Limeira, Brasil; b Applied Physiology and Nutrition Research Group – School of Physical Education and Sport and Faculdade de Medicina FMUSP, Universidade de São Paulo, São Paulo, Brazil; c Center of Lifestyle Medicine, Faculdade de Medicina FMUSP, Universidade de São Paulo, São Paulo, Brazil; d Nutrology Academy, Rio de Janeiro, Brazil

**Keywords:** Sports nutrition, ergogenic aid, cognitive performance, insomnia, Brazil

## Abstract

**Background:**

Electronic sports (esports) are a growing global phenomenon engaging millions of competitive players worldwide. Caffeine is a widely used compound for individuals seeking cognitive enhancement. However, evidence on consumption patterns, motivations, and safety in esports remains limited. We aimed to describe daily caffeine intake among Brazilian esports players and examine associations with competitive level, gaming habits, and adverse effects.

**Methods:**

Cross-sectional study of 181 Brazilian esports players. A 64-item questionnaire captured demographics, gaming habits, and caffeine intake from all dietary sources. We compared amateurs vs semi-professional/professional players and performance-motivated vs other motivations, and examined dose-response using intake categories (≤100, 101–300, 301–600, >600 mg/day) and correlations for continuous variables.

**Results:**

Median 168 mg/day (IQR 52–402; mean 280 ± 316); coffee was the main source (72.2% of total), and 55.8% consumed energy drinks, contributing 14.0% of intake. Overall, 25.7% exceeded 400 mg/day (46/179); intake did not differ between competitive levels (Amateur 172 vs Semi-Pro/Pro 121 mg/day; *p* = 0.387). No correlation with gaming hours (*ρ* = 0.068; *p* = 0.369). Under the primary positivity rule (≥“occasional”), adverse effects were common among respondents with symptom frequency data: any adverse effect 76.5%, insomnia 45.2%, tachycardia 29.1%, stomach pain 45.5%, tremors 23.7%. Linear trend tests across dose categories were not significant (any 0.822; insomnia 0.530; tachycardia 0.905; stomach pain 0.409; tremors 0.877), and per-category effect sizes were small (*r*-trend ≈ 0.01–0.08; OR per +1 category ≈ 0.89–1.16). Comparing >300 vs ≤300 mg/day for any adverse effect yielded OR 1.38 (95% CI 0.56–3.45). Performance-motivated players (12.6%) consumed more (+89 mg/day; *p* < 0.001). Using caffeine to combat fatigue (56.0%) was associated with higher insomnia (OR 2.50; 95% CI 1.37–4.55; *q* = 0.004). Notably, insomnia was common (45.2%), underscoring applied relevance.

**Conclusions:**

Brazilian esports players show moderate caffeine intake, mainly from coffee. Adverse effects were common, although linear dose-response across intake categories was not observed; the observed fatigue-caffeine cycle highlights the need for practical guidance on timing and source awareness, alongside sleep-hygiene strategies, to support sustainable performance.

## 1 Introduction

Electronic sports (esports) have transformed from niche entertainment into a multibillion-dollar global phenomenon, engaging millions of competitive players worldwide. Brazil has emerged as Latin America's esports powerhouse, with the country projected to achieve the region's highest compound annual growth rate from 2025 to 2030, driven by a passionate player base exceeding 90 million gamers and a thriving competitive ecosystem [1,2].

Within this intensely competitive digital arena, where milliseconds determine victory and cognitive performance (i.e. attention/focus, vigilance, and reaction time) is vital, caffeine is widely used by players seeking to enhance alertness, concentration, and reaction time during marathon gaming sessions that can extend beyond 12 hours [3,4]. A recent study of caffeine consumption patterns in Chinese elite esports athletes showed professional players consume an average of two large cups of coffee plus multiple energy drinks daily, roughly equivalent to ~150–200 mg per large coffee and ~80–160 mg per energy drink, representing the first consumption data in this rapidly growing population [5]. Coffee is a primary source of caffeine intake for several populations [6–8]. This emerging evidence highlights the critical need for a comprehensive understanding of caffeine use patterns in regions like Brazil where coffee culture intersects with competitive esports. Brazil is also the world's second-largest coffee consumer; recent Brazilian Coffee Industry Association (ABIC) data indicate rising consumption among 16–24-year-olds [9], reinforcing the relevance of examining caffeine use in this leading esports ecosystem.

Caffeine's ergogenic properties stem from its action as an adenosine receptor antagonist, with peak plasma concentrations achieved within 30–60 minutes of consumption and a half-life of 3–7 hours depending on individual metabolism [10]. Energy drinks typically contain 50–300 mg of caffeine per serving alongside other bioactive compounds such as taurine and B-vitamins [11], while additional caffeine sources, including soft drinks, supplements, chocolate, and tea, contribute to cumulative daily intake [6,7].

Caffeine's effects on cognitive performance, including enhanced attention, vigilance, and reaction time, align perfectly with the demands of competitive gaming [12–15]. The International Society of Sports Nutrition endorses 3–6 mg/kg body mass as the optimal ergogenic dose range, with higher doses (≥9 mg/kg) offering no additional benefits while substantially increasing the risk of adverse effects [16]. For cognition-oriented tasks, benefits typically occur toward the lower end of this range [17]. A meta-analysis in sports [18] and a broader systematic review focused on attention [19] reported significant improvements in attention accuracy (*g* = 0.27) and reaction time (*g* = 0.28) that may translate directly to gaming performance advantages. Indeed, Wu et al. (2024) demonstrated that 3 mg/kg caffeine significantly improved specific cognitive domains (shorter Stroop congruent reaction time and faster visual search reaction time with 20 items) and multiple shooting outcomes (higher kill ratio, higher hit accuracy, and shorter average time to target) in elite esports players [20]. Rogers et al. (2024) found that even low doses (1 mg/kg) enhanced first-person shooter performance [21], while Sainz et al. (2020) reported improved accuracy and reaction time in professional players [22]. These data indicate the benefit of caffeine consumption for esports performance.

Despite evidence to suggest caffeine may be beneficial to esports players [20–22], esports-specific consumption patterns remain limited, particularly with respect to gaming experience and adverse effects. Reporting of intake across all caffeine sources within the same cohort remains uncommon [5]. It would be particularly interesting to determine caffeine intake in Brazilian gaming populations considering the country's prominence in esports [1,2]. While caffeine can cause adverse effects, including anxiety, insomnia, tachycardia, and severe complications [23], data on consumption patterns and the incidence of these side effects in esports gamers are scarce. To our knowledge, no prior study has quantified caffeine intake or adverse effects in professional and non-professional Brazilian esports players.

Given the performance and financial stakes in professional esports, it remains unclear whether professional players consume more caffeine than amateurs and whether intake scales with time spent gaming. To address these gaps, the present study aimed to characterize habitual daily caffeine intake across all sources-as opposed to acute, session-specific dosing-among Brazilian esports players. We investigated correlations between intake patterns, competitive level, gaming habits, and adverse effects, and pre-specified four hypotheses: (H1) daily caffeine consumption is higher among semi-professional/professional versus amateur players; (H2) caffeine intake correlates positively with daily gaming hours; (H3) higher consumption associates with increased adverse effects (insomnia, tachycardia, stomach pain, tremors); and (H4) performance-motivated players consume more caffeine than those with other motivations.

## 2 Methods

### 2.1 Study design and participants

This cross-sectional study recruited Brazilian esports players through online gaming platforms and community forums between November 2023 and April 2024. From 303 initial respondents, 181 participants met the following inclusion criteria: age ≥ 18 years, self-identification as an esports player, Brazilian residence, and full completion of the study questionnaire. Competitive level was self-reported with three options (Amateur, Semi-professional, Professional). We categorized participants into two competitive levels: Amateur players (*n* = 146, 80.7%) and Semi-Professional/Professional players (*n* = 35, 19.3%), grouping semi-professional (*n* = 31) and professional (*n* = 4) a priori to ensure adequate statistical power for between-level comparisons. Sex categories comprised Men (*n* = 136, 75.1%), Women (*n* = 37, 20.4%), and Other/Non-binary (*n* = 5, 2.8%); three participants did not respond.

### 2.2 Ethical considerations

The Research Ethics Committee of the University of Campinas approved this study (CAAE: 53895021.6.0000.5404) in accordance with the Declaration of Helsinki guidelines [24]. All participants provided electronic informed consent before accessing the questionnaire. Data collection and storage protocols ensured complete participant anonymity, with all identifiers removed prior to analysis.

### 2.3 Data collection instrument

Participants completed a 64-item online questionnaire hosted on Respondi.app, designed to capture multidimensional aspects of caffeine consumption and gaming behavior. The instrument assessed: (1) sociodemographic characteristics including age, sex, education, and occupation; (2) gaming habits encompassing competitive level, primary game genres, average daily gaming hours, and years of experience; (3) detailed caffeine consumption patterns from all dietary sources (coffee, tea, energy drinks, soft drinks, chocolate, supplements) with frequency and quantity specifications; (4) occurrence and frequency of adverse effects potentially related to caffeine; and (5) motivations underlying caffeine use. Motivations were captured via a multiple-response item (checkboxes), allowing participants to select more than one reason; the option “to combat fatigue or avoid drowsiness” was presented as a single combined item. No Likert scale was applied to motivations. The complete questionnaire is available as Supplementary Material A. Each motivation was analyzed as a binary variable (selected vs. not selected), permitting multiple simultaneous selections. The combined “to combat fatigue or avoid drowsiness” item was treated as a single indicator; for the caffeine-fatigue vicious cycle analysis, this indicator was used to define comparison groups.

### 2.4 Data processing and analysis

#### 2.4.1 Caffeine quantification

Daily caffeine intake was calculated using a computational algorithm that processed questionnaire responses for all reported sources. The algorithm incorporated caffeine content values derived from the Brazilian Food Composition Table (TACO) [25], international food composition databases [26], and peer-reviewed literature on caffeine content in Brazilian products [8]. These reference values, representing typical caffeine concentrations in Brazilian products, were compared across multiple databases to promote consistency. The algorithm converted weekly consumption frequencies to daily intake using the formula:

DailyCaffeine(mg)=CaffeineContentperUnit×Volume/WeightConsumed×DailyFrequencyMultiplier

Total daily intake represented the sum of caffeine from all reported sources. The complete reference table is provided in Supplementary Material B. As body weight data were not collected, only absolute intake (mg/day) could be calculated, precluding relative dose assessments.

#### 2.4.2 Adverse effects assessment

**Figure 1.**
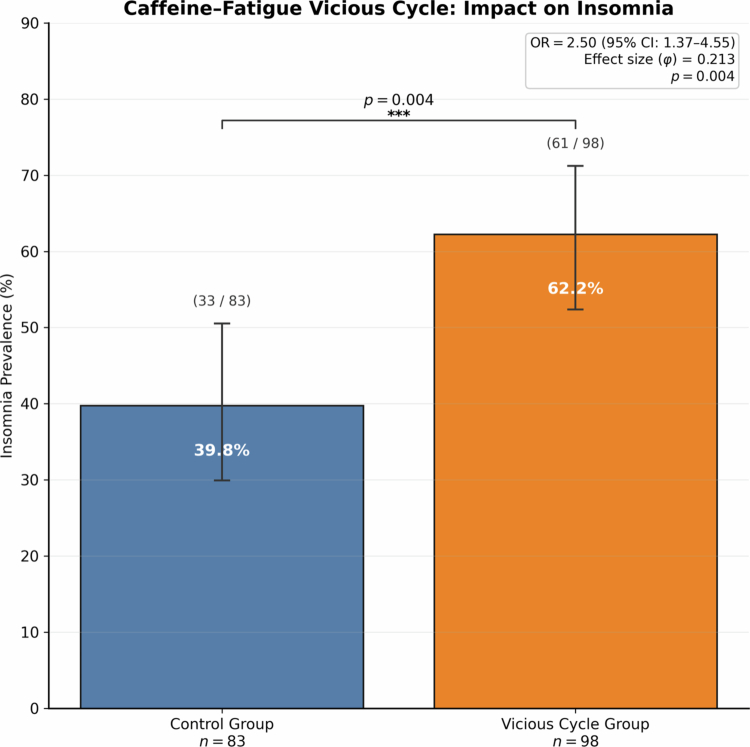
Caffeine-fatigue vicious cycle: impact on insomnia. Bars show insomnia prevalence (95% Wilson CIs) for Control (*n* = 83) vs Vicious Cycle group (*n* = 98); insomnia defined as ≥ “rarely”. χ² = 8.234, *p* = 0.004; OR = 2.50 (95% CI: 1.37–4.55); effect size *φ* = 0.213.

Participants reported the frequency of experiencing specific symptoms potentially related to caffeine consumption (insomnia, tachycardia, tremors, stomach pain) on a five-point Likert scale ranging from “never” to “always.” For primary analyzes, we defined "presence" using ≥ “occasional” (excluding “rarely”), consistent with epidemiologic practice; sensitivity analyzes used (i) ≥ “rarely” and (ii) ≥ “frequent” (combining “frequently” + “always”). For the caffeine-fatigue vicious cycle analysis displayed in Figure 1, insomnia was defined a priori as ≥ “rarely” to capture any complaint frequency. For dose-response analyzes, participants were categorized into four intake groups: Low (≤100 mg/day), Moderate (101–300 mg/day), High (301–600 mg/day), and Very High (>600 mg/day). These dose bands were defined a priori considering widely used limits (e.g. EFSA 2015 [27] and ≥400 mg/day as high consumption) and the sample distribution. As additional sensitivity analyzes, we evaluated correlations between continuous dose and frequency of adverse events and contrasted >300 vs ≤300 mg/day.

#### 2.4.3 Statistical analysis

We conducted all analyzes using Python 3.9 with pandas for data manipulation [28], scipy.stats for statistical tests, and scikit-posthocs for post-hoc comparisons. Given the non-normal distribution of caffeine consumption data (Shapiro-Wilk test *p* < 0.001), we employed non-parametric methods throughout. Mann-Whitney U tests compared caffeine intake between independent groups (H1, H3, H4), with effect sizes calculated as *r* = Z/√n. Spearman's rank correlation assessed correlations between continuous variables (H2). Multiple comparison control followed a family-wise false discovery rate (BH-FDR, *q* = 0.05) [29] applied within logically defined families: (i) H3 comprises four primary outcomes (insomnia, tachycardia, stomach pain, tremors) for which BH-FDR was applied across the four *p*-values; (ii) H1, H2, and H4 each contained a single primary test and therefore did not require multiplicity correction. Tables denote where *p*-values are FDR-adjusted. For exploratory analyzes, we conducted a post-hoc power analysis revealing Cohen's *d* = 0.35 and achieved power = 0.78 for detecting differences between competitive levels (*α* = 0.05). Multiple linear regression on log-transformed caffeine consumption examined predictors including gaming hours, performance intention, competitive level, and sex. Cochran-Armitage trend tests evaluated dose-response relationships for adverse effects. Odds ratios (OR) with 95% confidence intervals compared risk of adverse effects between high-dose (>300 mg/day) and low-dose (≤300 mg/day) groups. Effect-size thresholds followed established conventions: Cohen's d (small = 0.20, medium = 0.50, large = 0.80) and rank correlations r or *ρ* (small = 0.10, medium = 0.30, large = 0.50); rank-biserial correlation used the same r thresholds [30,31]. We calculated 95% confidence intervals where applicable (e.g. odds ratios and binomial proportions); otherwise we report point estimates and *p*-values.

**Figure 2.**
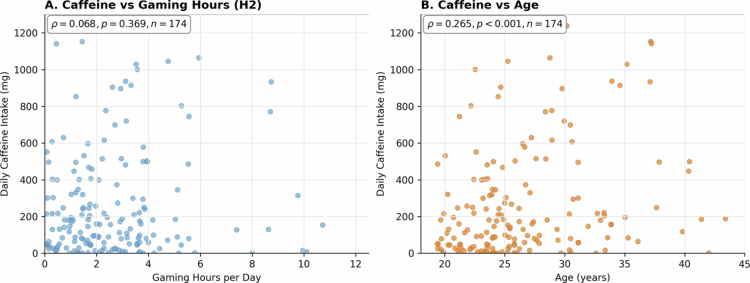
Correlation analyzes. Panel A: Caffeine vs Gaming Hours (H2). Spearman *ρ* = 0.017, *p* = 0.823, *n* = 174. Panel B: Caffeine vs Age. Spearman *ρ* = 0.265, *p* < 0.001, *n* = 174.

Primary analyzes used an Analytic Data Set (ADS; *N* = 179) created a priori by applying quality filters, including exclusion of implausible daily intakes >2000 mg/day (*n* = 2). Unless stated otherwise, results refer to the ADS. For Figure 2 (correlations), we used a unified complete-case sample across both panels so that both analyzes report the same *n* (*n* = 174) based on simultaneous availability of caffeine intake, daily gaming hours, and age.

Given the pronounced right-skew and extreme values in daily caffeine intake, we report median (IQR) as the primary descriptive statistic. Primary analyzes used an analytic dataset (ADS) with pre-specified quality filters, yielding *N* = 179. As sensitivity analyzes, we also computed a 10% trimmed mean and a winsorized mean with an upper cap of 1000 mg/day on the unfiltered distribution to assess robustness.

## 3 Results

### 3.1 Participant characteristics

The study sample comprised 181 Brazilian esports players with a mean age of 22 years (range: 18–45 years). The majority were men (75.1%, *n* = 136), with 80.7% (*n* = 146) competing at amateur level and 19.3% (*n* = 35) at semi-professional/professional level. Primary game genres included first-person shooters (FPS, 37.0%), multiplayer online battle arenas (MOBA, 25.4%), and sports simulations (11.6%). Participants reported a median of 2.0 hours/day (IQR: 1.0–4.0). Semi-professional/professional players reported significantly higher daily gameplay compared to amateurs (Median 3.0 vs 1.5 hours; *p* < 0.001). No significant differences were observed between competitive levels for age, sex distribution, or primary gaming platform (all *p* > 0.05). Table 1 presents complete sociodemographic and gaming characteristics stratified by competitive level.

### 3.2 Caffeine consumption patterns

**Figure 3.**
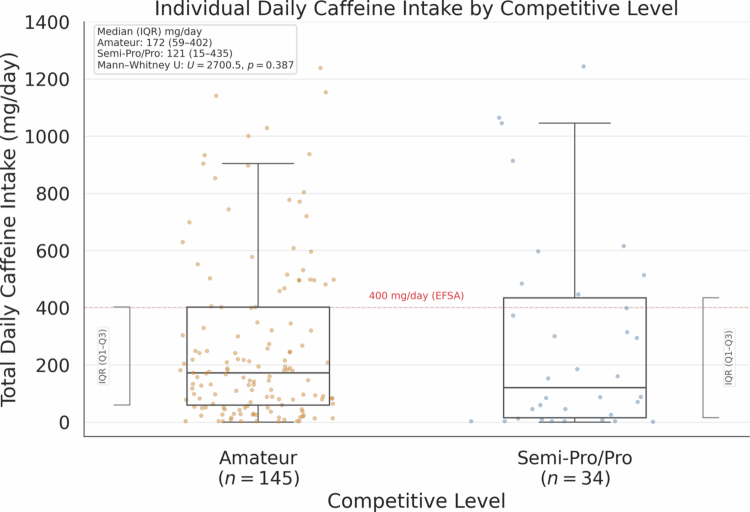
Individual daily caffeine intake (mg/day) by competitive level. Boxplots show median and interquartile range (IQR); whiskers extend to values within ±1.5 × IQR and points beyond represent outliers. The dashed line marks 400 mg/day (EFSA). No significant difference between groups (Mann-Whitney *U* = 2700.5, *p* = 0.387). Amateur: 172 mg/day (IQR 59–402; *n* = 145). Semi-Pro/Pro: 121 mg/day (IQR 15–435; *n* = 34). Note: Figure computed on the ADS (*N* = 179; excludes two >2000 mg/day outliers); Table 1 describes the full sample (*N* = 181).

Daily caffeine consumption under ADS (*N* = 179) demonstrated substantial variability, with a median intake of 168 mg/day (IQR: 52–402 mg/day) and a mean of 280 ± 316 mg/day. A small subset reported no habitual caffeine consumption (*n* = 4; 2.2% of the full sample). Overall, 25.7% exceeded 400 mg/day (46/179). Contrary to our primary hypothesis (H1), competitive level did not significantly influence caffeine intake: amateur players consumed a median of 172 mg/day compared to 121 mg/day among semi-professional/professional players (Mann-Whitney U = 2700.5, p = 0.387, *r* = 0.065). Figure 3 displays the distribution by competitive level. Note that the figure represents the ADS (*N* = 179), whereas Table 1 includes the full sample (*N* = 181). This null finding persisted after adjusting for age and gaming hours in regression analyzes. Gaming duration showed no association with caffeine intake (H2), with Spearman correlation revealing ρ = 0.068 (*p* = 0.369) (Figure 2A). Significant associations emerged between caffeine intake and certain adverse effects (H3), namely insomnia (*U* = 2,847, *q* = 0.015, *r* = 0.18) and stomach pain (*U* = 2,156, *q* = 0.009, *r* = 0.19), after BH-FDR correction within the four H3 outcomes. There was no association between caffeine intake and tachycardia or tremors (Table 2). Players reporting performance enhancement as a motivation for caffeine consumption demonstrated significantly higher consumption (H4), with a difference in medians of 89 mg/day compared to other motivations (*U* = 1,523, *p* < 0.001, *r* = 0.28). Table 2 provides results for all hypotheses tested with effect sizes.

### 3.3 Caffeine sources and cultural patterns

Coffee emerged as the dominant caffeine source among Brazilian esports players, contributing 72.2% of total daily intake. Among all participants, 76.2% (n = 138) reported regular coffee consumption. Energy drinks represented the second largest source (14.0% of total intake), consumed by 55.8% of participants with a mean contribution of 74.7 mg/day of caffeine among users. Other sources included caffeinated soft drinks (5.1%), supplements (3.8%), chocolate (3.2%), and tea (1.7%). Participants typically combined multiple sources, consuming caffeine from an average of 3.2 different sources daily (median = 3, range: 0–6). Competitive level did not significantly influence source preferences: coffee consumption rates did not differ significantly between amateur (80.8%) and semi-professional/professional players (74.3%, p = 0.410), nor did energy drink use patterns (45.9% vs 40.0%, p = 0.538).

**Figure 4.**
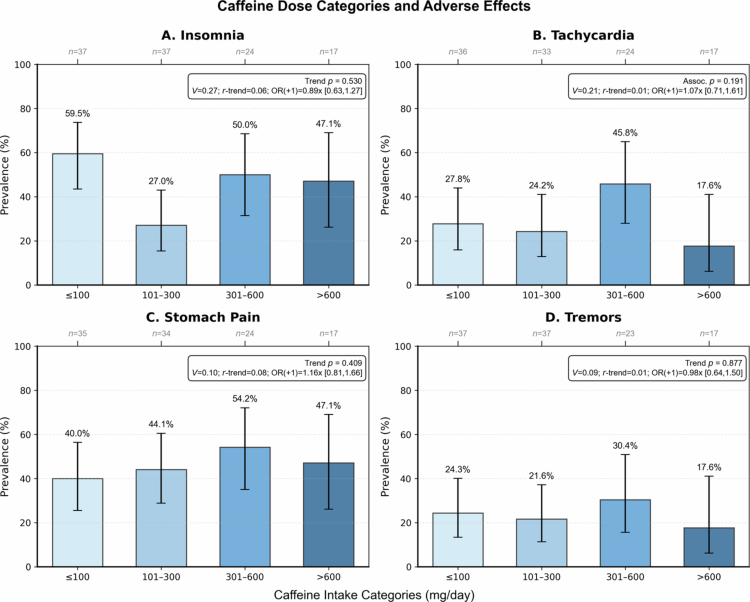
Dose-response relationships between caffeine intake categories and adverse effects prevalence (A: Insomnia; B: Tachycardia; C: Stomach pain; D: Tremors). Categories: ≤100, 101–300, 301–600, >600 mg/day. Error bars show 95% Wilson CIs. Primary positivity threshold is ≥ “occasional.” Panel annotations report Cramér's V (*φ*), *r*-trend, and OR per +1 category with 95% CI; Cochran-Armitage trend tests were not significant in any panel (see *p*-values in annotations).

Under the primary positivity rule (≥ “occasional”), adverse effects were common among respondents with symptom frequency data: any adverse effect 76.5%, insomnia 45.2%, tachycardia 29.1%, stomach pain 45.5%, tremors 23.7%. Linear trend tests across dose categories were not significant (any 0.822; insomnia 0.530; tachycardia 0.905; stomach pain 0.409; tremors 0.877), and per-category effect sizes were small (r-trend ≈ 0.01–0.08; OR per +1 category ≈ 0.89–1.16). Comparing >300 vs ≤300 mg/day for any adverse effect yielded OR 1.38 (95% CI 0.56–3.45). Several panels showed non-monotonic patterns (e.g. higher prevalence at 301–600 mg/day but not at >600 mg/day), which may reflect tolerance or selection effects among very high consumers. Prevalence by intake category is summarized in Table 3, and dose-response patterns are shown in Figure 4.

### 3.4 Motivations and performance enhancement

Participants reported diverse motivations for caffeine consumption, with multiple reasons often coexisting. The most prevalent motivation was combating fatigue or avoiding drowsiness (56.0%, *n* = 98). To explore the clinical implication of this primary motivation, we examined the association between using caffeine “to combat fatigue or avoid drowsiness” and the prevalence of insomnia. This analysis revealed that participants in this “Vicious Cycle Group” (*n* = 98) had 2.5 times the odds of reporting insomnia compared to the control group (*n* = 83), a statistically significant finding (OR 2.50; 95% CI 1.37-4.55; *p* = 0.004), as detailed in (Figure 1). Following this, other common motivations included pleasure/taste appreciation (53.1%, *n* = 93), habitual/routine consumption (49.1%, *n* = 86), concentration enhancement (37.1%, *n* = 65), and specific gaming performance improvement (12.6%, *n* = 22). As detailed in the Methods (Section 2.3), the questionnaire item for motivations allowed multiple selections (checkboxes), and “to combat fatigue or avoid drowsiness” was presented as a single, combined option. While only 12.6% explicitly cited gaming performance as a primary motivation, these individuals consumed significantly more caffeine than all other respondents combined (comparison: participants who endorsed “performance” vs. all other motivations combined), supporting H4 (Table 2). Correlation analyzes showed no relationship between caffeine intake and daily gaming hours (Figure 2A; ρ = 0.017, *p* = 0.823; *n* = 174), whereas age demonstrated a weak positive correlation with intake (Figure 2B; ρ = 0.265, *p* < 0.001; *n* = 174). In a multiple linear regression of log-transformed intake including gaming hours, performance intention, competitive level, and sex, the model explained ~11% of variance (adjusted *R*² = 0.108; *F* (4, 176) = 6.41, *p* < 0.001). Two predictors were significant: performance intention (higher intake; beta = 1.151, *p* = 0.001) and semi-professional/professional status (lower intake; beta = −0.689, *p* = 0.042).

**Table 1.** Sociodemographic and gaming characteristics of participants by competitive level.

Characteristic	Amateur (*n* = 146)	Semi-Professional/Professional (*n* = 35)	Total (*N* = 181)	*p*-value
Age (years), mean ± SD	22.1 ± 4.8	23.8 ± 5.2	22.4 ± 4.9	0.089
**Sex,** *n* **(%)**	-	-	-	0.353
Men	107 (73.3)	29 (82.9)	136 (75.1)	-
Women	33 (22.6)	4 (11.4)	37 (20.4)	-
Other	4 (2.7)	1 (2.9)	5 (2.8)	-
Not reported	2 (1.4)	1 (2.9)	3 (1.7)	-
Gaming hours/day, median (IQR)	1.5 (1.0–3.0)	3.0 (2.0–5.5)	2.0 (1.0–4.0)	<0.001*
**Primary platform,** *n* **(%)**	-	-	-	0.534
PC	89 (61.0)	22 (62.9)	111 (61.3)	-
Console	45 (30.8)	9 (25.7)	54 (29.8)	-
Mobile	12 (8.2)	4 (11.4)	16 (8.8)	-
**Primary genre,** *n* **(%)**	-	-	-	0.623
FPS	52 (35.6)	15 (42.9)	67 (37.0)	-
MOBA	38 (26.0)	8 (22.9)	46 (25.4)	-
Battle Royale	28 (19.2)	7 (20.0)	35 (19.3)	-
Sports	18 (12.3)	3 (8.6)	21 (11.6)	-
Other	10 (6.8)	2 (5.7)	12 (6.6)	-

Abbreviations: FPS, first-person shooter; MOBA, multiplayer online battle arena. *p < 0.05. Statistical tests: Mann-Whitney U test for continuous variables, Chi-square test for categorical variables. Note: Table 1 reports the full sample (*N* = 181). Other analyzes-such as Figure 1-use the ADS (*N* = 179) after excluding two >2000 mg/day outliers.

## 4 Discussion

This study aimed to characterize caffeine consumption patterns among Brazilian esports players and examine associations with gaming habits, competitive level, and adverse effects. Our analytic dataset (*N* = 179; ADS after removing two >2000 mg/day outliers) demonstrated moderate caffeine intake levels (median 168 mg/day; IQR 52–402; mean 280 ± 316), comparable to the general U.S. population average (165 mg/day) [6], and within the range reported for Brazilian competitive athletes. The similarity in consumption between amateur and semi-professional/professional players challenges assumptions about expertise-driven intake patterns, suggesting that competitive level alone does not determine caffeine use behaviors in esports players. Our findings unveil a complex risk-benefit scenario, where caffeine's cognitive enhancement potential coexists with potential adverse effects; notably, we did not observe significant linear dose-response trends across intake categories.

Our exploratory analysis provides suggestive evidence consistent with a caffeine-fatigue cycle, affecting 56.0% of Brazilian esports players. Our data on insomnia prevalence among those using caffeine to combat fatigue aligns with findings in healthcare professionals linking higher caffeine use, poorer sleep quality, and burnout [9], and with experimental evidence that caffeine consumed 0 to 6 hours before bedtime impairs sleep [32–35], representing a novel observation in competitive gaming contexts. This self-perpetuating cycle, where caffeine disrupts sleep, poor sleep increases fatigue, and fatigue drives further caffeine consumption, poses particular concerns for young adults establishing potentially lifelong consumption patterns. The identification of this phenomenon in a population with a mean age of approximately 22 years (22 ± 5 years) suggests the need for early interventions to prevent chronic sleep-caffeine dysregulation.

Cultural factors may shape these consumption patterns. As the world's second-largest coffee consumer, Brazil's deeply embedded coffee culture manifests clearly in our data: 76.2% of players regularly consume coffee, contributing 72.2% of total caffeine intake. This coffee dominance contrasts sharply with international gaming populations, in which energy drinks often predominate [5]. Recent data from the Brazilian Coffee Industry Association shows a 7.47% consumption growth among 16–24 year-olds, ten times higher than other age groups [9], suggesting that this pattern will likely intensify. Compared to U.S. college students [36], Brazilian esports players showed similar prioritization of alertness and pleasure as primary motivations. Moreover, our sample's mean intake (280 mg/day) is higher than the 234 mg/day reported for young adults (≥23 years) [36]. Notably, German esports players reported minimal energy drink consumption [37], while our Brazilian sample showed substantially higher energy drink intake alongside traditional coffee consumption. The cultural normalization of coffee consumption may explain why Brazilian esports players maintain moderate overall intake despite high participation rates.

Taken together, these results do not support a linear, monotonic dose-response across intake categories; observed effects were small and confidence intervals were wide. In practice, caffeine's cognitive benefits in esports are most consistent at moderate doses when used judiciously and with attention to timing. Evidence from esports-specific trials indicates performance benefits with approximately 1–3 mg/kg [20–22]. Conversely, relying on caffeine primarily to “combat fatigue” late in the day may increase insomnia risk and perpetuate the caffeine-sleep cycle; avoiding intake within roughly 6 hours of planned sleep is advisable [32–35].

In our multivariable model of log-transformed intake, predictors collectively explained ~11% of variance (adjusted *R*² = 0.108). Performance intention was associated with higher intake, whereas semi-professional/professional status was associated with lower intake when controlling for other covariates. Although exploratory and not causal, this pattern suggests that intention-not competitive level per se-better captures consumption behavior and underscores the practical need to balance potential cognitive benefits against adverse effects through careful dose and timing.

From a public health perspective, approximately one-quarter of participants exceeded 400 mg/day (25.7%; 46/179) in the ADS, a commonly cited threshold for high consumption in adults. While our dose-category trend tests did not support a linear, monotonic increase in adverse effects and several panels suggested non-monotonic patterns, this subgroup nonetheless warrants attention. In settings without widely disseminated national caffeine guidance, practical advice emphasizing conservative dosing, dose-timing, and source awareness may help mitigate risk among heavier consumers.

This study's comprehensive approach, integrating multiple caffeine sources with Brazilian reference tables, employing robust statistical methods with effect size reporting, and achieving adequate statistical power (0.78), provides a solid methodological foundation. The novel quantification of the vicious cycle phenomenon and detailed dose-response relationships offer clinically actionable insights. This study highlights the need for evidence-based educational guidelines emphasizing: (1) safe dosing (200–400 mg) taken 30–60 minutes before competition [10], (2) source awareness to prevent excessive intake, and (3) integration within broader sleep hygiene and nutrition frameworks to minimize adverse effects.

However, several limitations warrant consideration. The cross-sectional design precludes causal inferences, while self-reported data may introduce recall bias. Our convenience sampling through online communities could attract participants with stronger opinions about caffeine or those experiencing adverse effects, potentially inflating prevalence estimates. The absence of body weight data prevented calculation of relative doses (mg/kg), limiting comparison with sports nutrition guidelines. We did not measure timing of intake relative to competitions or sleep. Additionally, novel caffeine sources like gums and medications were not captured. Importantly, caffeine content in many sources varies substantially across brands, preparations, and serving sizes [8,38,39], introducing additional imprecision into intake estimation. The study did not account for genetic variations (e.g. CYP1A2) that influence caffeine metabolism [40]. Recent Brazilian research has demonstrated that genetic polymorphisms, including ACE gene variations, may affect caffeine response in athletic populations [40,41].

Future research should employ longitudinal designs to establish temporal relationships between caffeine use, sleep quality, and performance outcomes. Genetic testing for caffeine metabolism variants could explain individual response variations, while objective sleep monitoring would validate self-reported insomnia. Qualitative studies exploring beliefs and social influences shaping consumption patterns would complement quantitative findings. Timing of intake in relation to competition and sleep should be evaluated to inform event-day guidance. The prevalence of energy drink sponsorships in esports also warrants investigation regarding marketing influences on young consumers. Beyond traditional PC/console/mobile titles, parts of the esports ecosystem increasingly include physically interactive “virtual sports”, formally recognized in the International Olympic Committee's Olympic Esports Series 2023 [42]. These developments reinforce the relevance of sports nutrition to esports; future investigations should evaluate how ergogenic aids such as caffeine influence both cognitive and physiological outcomes in these hybrid competitive environments.

### 4.1 Practical implications

For esports players seeking cognitive benefits while minimizing adverse effects, practical guidance includes:

- Esports-specific education: communicate the potential benefits for cognitive tasks with ~1–3 mg/kg, with emphasis on timing and the minimal effective dose [20–22].
- Prefer relative dosing: approximately 1–3 mg/kg is often sufficient for cognitive tasks, generally lower than exercise-focused recommendations [16,17].
- Avoid intake within ~6 hours of planned sleep to reduce insomnia risk [33–35].
- Track cumulative intake across sources (coffee, energy drinks, soft drinks, chocolate, supplements) and start with the lowest effective dose.
- Consider individual variability in response and tolerance; adjust timing and dose conservatively.

**Table 2.** Caffeine consumption patterns and hypothesis testing results.

Hypothesis	Variable	Test Result	*p* (FDR for H3)	Effect Size
H1	Daily caffeine by competitive level	Amateur: 172 mg; Semi-Pro/Pro: 121 mg; ***U*** = 2700.5	0.387	*r =* 0.065 (negligible)
H2	Caffeine vs gaming hours correlation	*ρ* = 0.017 (no correlation)	0.823	−
H3	Caffeine vs adverse effects- Insomnia	*U* = 2,847	0.015*	*r =* 0.18 (small)
H3	Caffeine vs adverse effects- Stomach pain	*U* = 2,156	0.009*	*r =* 0.19 (small)
H3	Caffeine vs adverse effects- Tachycardia	*U* = 3,124	0.082	*r =* 0.13
H3	Caffeine vs adverse effects- Tremors	*U* = 3,287	0.156	*r =* 0.11
H4	Performance motivation vs intake	Median difference: 89 mg/day higher; *U* = 1,523	<0.001*	*r =* 0.28 (small-medium)

^*^ Statistical tests: Mann-Whitney U test for group comparisons between amateur and semi-professional/professional players, Spearman correlation for continuous variables. Asterisks (*) denote p < 0.05 (statistically significant) using the appropriate approach: for H3 rows, p-values are BH-FDR adjusted at q = 0.05 across the four H3 outcomes; for H1, H2, and H4, p-values are unadjusted. Effect sizes: r = Z/√n for Mann-Whitney U tests.

**Table 3.** Prevalence of self-reported adverse effects by caffeine dose categories.

Adverse Effect	Low (≤100 mg)	Moderate (101–300 mg)	High (301–600 mg)	Very High (>600 mg)	*p*-value*	OR (95% CI)†
Insomnia	22/37 (59.5%)	10/37 (27.0%)	12/24 (50.0%)	8/17 (47.1%)	0.530	1.25 (0.58–2.66)
Tachycardia	10/36 (27.8%)	8/33 (24.2%)	11/24 (45.8%)	3/17 (17.6%)	0.905	1.47 (0.64–3.36)
Tremors	9/37 (24.3%)	8/37 (21.6%)	7/23 (30.4%)	3/17 (17.6%)	0.877	1.13 (0.47–2.73)
Stomach pain	14/35 (40.0%)	15/34 (44.1%)	13/24 (54.2%)	8/17 (47.1%)	0.409	1.44 (0.67–3.11)
Any adverse effect	31/37 (83.8%)	24/37 (64.9%)	20/24 (83.3%)	13/17 (76.5%)	0.822	1.38 (0.56–3.45)

Note: Denominators represent only respondents who provided frequency data for each specific symptom. The apparent non-linear dose-response for some adverse effects, where prevalence plateaued or decreased in the highest intake category (>600 mg/day, small n), may reflect pharmacological tolerance or selection effects among habitual heavy consumers.

^*^ Cochran-Armitage test for linear trend. †Odds ratio comparing the combined high-dose groups (>300 mg/day total) vs. the combined low-dose groups (≤300 mg/day total).

## 5 Conclusions

Brazilian esports players demonstrated moderate caffeine consumption patterns dominated by coffee, with dose-related adverse effects. Findings challenge assumptions about higher intake at advanced competitive levels and highlight a potential caffeine-fatigue vicious cycle in young players. These results underscore the need for evidence-based guidance tailored to esports contexts and further research on timing, dose-response, and individual variability.

## Data Availability

The dataset analyzed during the current study is available from the corresponding author upon reasonable request.
